# Oral Samples as Non-Invasive Proxies for Assessing the Composition of the Rumen Microbial Community

**DOI:** 10.1371/journal.pone.0151220

**Published:** 2016-03-17

**Authors:** Ilma Tapio, Kevin J. Shingfield, Nest McKain, Aurélie Bonin, Daniel Fischer, Ali R. Bayat, Johanna Vilkki, Pierre Taberlet, Timothy J. Snelling, R. John Wallace

**Affiliations:** 1 Green Technology, Natural Resources Institute Finland, Jokioinen, Finland; 2 Rowett Institute of Nutrition and Health, University of Aberdeen, Aberdeen, United Kingdom; 3 Laboratoire d'Ecologie Alpine, CNRS, Grenoble, France; Agriculture and Agri-Food Canada, CANADA

## Abstract

Microbial community analysis was carried out on ruminal digesta obtained directly via rumen fistula and buccal fluid, regurgitated digesta (bolus) and faeces of dairy cattle to assess if non-invasive samples could be used as proxies for ruminal digesta. Samples were collected from five cows receiving grass silage based diets containing no additional lipid or four different lipid supplements in a 5 x 5 Latin square design. Extracted DNA was analysed by qPCR and by sequencing 16S and 18S rRNA genes or the fungal ITS1 amplicons. Faeces contained few protozoa, and bacterial, fungal and archaeal communities were substantially different to ruminal digesta. Buccal and bolus samples gave much more similar profiles to ruminal digesta, although fewer archaea were detected in buccal and bolus samples. Bolus samples overall were most similar to ruminal samples. The differences between both buccal and bolus samples and ruminal digesta were consistent across all treatments. It can be concluded that either proxy sample type could be used as a predictor of the rumen microbial community, thereby enabling more convenient large-scale animal sampling for phenotyping and possible use in future animal breeding programs aimed at selecting cattle with a lower environmental footprint.

## Introduction

Ruminant livestock production has a large environmental footprint because of emissions of the greenhouse gas, methane, and to high nitrogenous emissions in urine and faeces [1]. The primary source of these emissions is rumen microbial metabolism [2]. Thus, understanding the ruminal microbiota is a vital prerequisite for improving the environmental credentials of meat and milk production. The introduction of high-throughput sequencing techniques has opened new ways to explore complex microbial ecosystems, including the rumen [3–6]. Sampling ruminal digesta is essential to enable the technology benefits to be realized. Rumen sampling can be carried out by oral intubation, but this is an unpleasant procedure for the animal and also results in a sample that is often heavily contaminated with saliva [7]. Rumenocentesis provides valid samples but involves puncturing the abdominal wall with a needle and removing digesta by syringe, also undesirable in terms of animal welfare [8], and restricts the amount of sample that can be collected. The most reliable samples are obtained from animals that have been surgically modified by fitting a ruminal cannula [7,9], but this requires skilled surgery, dedicated animal facilities and in most countries requires formal governmental permission. In any case, rumen fistulation is impractical for sampling large numbers of animals.

Ruminants regurgitate ruminal contents regularly in order to chew the partially digested plant material [10,11]. The chewed bolus is then swallowed for further microbial degradation. It might be expected, therefore, that the microbiome of the mouth could represent a reflection of the ruminal microbiome. If so, collection of small samples of oral fluid could be used as a proxy for assessing the microbial ecology of the rumen, avoiding the need for more invasive sampling procedures. Our hypothesis was that the oral sample will contain microbes from the regurgitated bolus that results from rumination and that the microbial composition of the bolus might be representative of the ruminal community. Another proxy that has been investigated before is faeces. The faecal community is significantly different to that of the rumen [12–15], but nonetheless there may be indicators that could prove useful, analogous to the presence of faecal archaeol, a membrane lipid of ruminal archaea, being used as a marker for ruminal methanogenesis [16–18]. The aim of the present experiment was to compare the communities in these potential alternative samples in order to evaluate their usefulness as proxies for direct sampling of ruminal digesta. A paper has been published recently [19] in which the same hypothesis was explored using buccal samples in sheep. The present paper confirms the conclusions of that paper concerning the validity of buccal sampling, in this case using dairy cows, and further amplifies the investigation by comparing bolus and faecal samples.

## Materials and Methods

### Animal experimentation

All experimental procedures were approved by the National Ethics Committee (Hämeenlinna, Finland) in accordance with the guidelines established by the European Community Council Directives 86/609/EEC [20]. The experiment was conducted between 15 February 2012 and 3 July 2012 at Natural Resources Institute Finland (formerly MTT Agrifood Research Finland), Jokionen Finland (60.8° N, 23.5° E; altitude 103 m). Five Finnish Ayrshire cows fitted with rumen cannula (#1C, i.d. 100 mm, Bar Diamond, Inc., Parma, ID) of (mean ± SE) 4 ± 0.6 parity, 63 ± 11.7 d in milk, and 705 ± 25.5 kg live weight were used in a 5 × 5 Latin square with 28-d experimental periods. Treatments comprised total mixed rations based on grass silage (forage: concentrate ratio 60:40 on a dry matter (DM) basis) containing no additional fat (CO) or 50 g/kg diet DM of methyl esters of myristic acid (MA), rapeseed oil (RO), safflower oil (SO) or linseed oil (LO). Lipid supplements replaced concentrate ingredients. Each period comprised 2 d adaptation, 21 d supplementation, and 5 d washout to minimize treatment carry-over effects. Samples for the analysis of rumen microbial composition were collected at 15.00 h on d 20 and 09.00 h on d 22 of each period.

### Collection and processing of samples

Ruminal digesta samples were collected from four regions (anterior dorsal, anterior ventral, posterior dorsal, and posterior ventral) within the rumen-reticulum. Immediately after collection, ruminal digesta samples were mixed thoroughly and squeezed through 2 layers of cheesecloth. Five hundred μl of rumen liquid were mixed with 1 ml of phosphate buffered saline-glycerol (30% v/v) buffer (PBS-gly) and immediately frozen at -80°C.

Regurgitated ingesta (bolus) samples were collected as close in time as possible to that of rumen samples. Depending on the rumination behaviour of each cow, the time after rumen sampling varied among the animals but did not exceed 20 min. Bolus samples were processed in the same way as rumen samples.

Buccal samples (effectively saliva mixed with bolus particles) were collected at the same time as bolus using sponge swabs. Three collection methods were investigated: (i) samples were collected using the BuccalAmp DNA extraction kit (Epicentre) and (ii) using the Performagene Livestock (PG-100) kit (DNA genotek) and processed following the manufacturer’s protocols, or (iii) samples were collected using Performagene Livestock sponge swabs, immediately submerged in 1 ml of PBS-gly buffer and frozen at -80°C. Processing of samples based on method (iii) was the best in terms of DNA quality and quantity, and therefore used for sampling. For technical reasons, buccal swabs could not be taken during the first experimental period.

Fresh faeces were collected by stimulating rectal activity at the time around rumen sampling. Twenty five g of faeces were preserved in 50 ml of PBS-gly buffer and stored at -80°C.

Total genomic DNA was extracted from 1 ml of mixed d 20 and d 22 sample (in case of rumen, bolus and buccal swab) or 30 mg of faeces following the protocol of Yu and Morrison [21].

### Quantitative PCR of 16S and 18S rRNA genes

DNA concentrations were determined with a NanoDrop ND 1000 Spectrophotometer (NanoDrop Technologies, Wilmington, DE). DNA was diluted to 0.1 ng/μl in 5 μg/ml herring sperm DNA for amplification with universal bacterial primers UniF and UniR [22] and 1 ng/μl in 5 μg/ml herring sperm DNA for amplification of other groups [23]. Quantitative PCR was carried out using a BioRad CFX96 as described by Ramirez-Farias et al. [24]. Primer sets and target species are described in Table A in S1 Text. Amplification of archaeal 16S RNA genes was carried out using the primers described by Hook et al. [25] and calibrated using DNA extracted from *Methanobrevibacter smithii* PS, a gift from M. P. Bryant, University of Illinois. Amplification efficiency was evaluated using template DNA from *Roseburia hominis* A2-183 (DSM 16839^T^) for the universal bacteria and *Clostridium* Cluster XIVa calibrations, *Faecalibacterium prauznitzii* A2-165 (DSM 17677^T^) for *Clostridium* Cluster IV, and *Bacteroides thetaiotaomicron* VPI-5482 (DSM 2079^T^) for Bacteroidetes. Protozoal 18S rRNA gene amplification was calibrated using DNA amplified from bovine rumen digesta with primers 54f and 1747r [26]. Coverage of qPCR primers was checked from original references and by use of the Probe Match tool of the Ribosome Database Project [27]. Bacterial abundance was calculated from quadruplicate Ct values using the universal bacterial calibration equation. Pairwise differences between treatments and between samples within treatments were evaluated by a simple t-test. *P* values have been adjusted for multiple testing using the Benjamini-Hochberg method [28].

### Amplicon preparation and sequencing

Primers used for PCR amplification of bacteria and archaea 16S rRNA genes, ciliate protozoa 18S rRNA genes and anaerobic fungi ITS1 genes were designed in silico using ecoPrimers [29], the OBITools software suite [30] (http://www.grenoble.prabi.fr/trac/OBITools) and a database created from sequences stored in GenBank. For each sample, PCR amplifications were performed in duplicate. An eight nucleotide tag unique to each PCR duplicate was attached to the primer sequence, in order to enable the pooling of all PCR products for sequencing and the subsequent assignation of sequence reads to their respective samples. PCR amplicons were combined in equal volumes and purified (QIAquick PCR purification kit, Qiagen, Germany). Amplicon libraries were prepared in Fasteris SA (Geneva, Switzerland https://www.fasteris.com/dna/) using the TruSeq Nano DNA HT Sample Prep Kit from Illumina using a protocol with only five PCR cycles (https://www.fasteris.com/dna/?q=content/metafast-protocol-amplicon-metagenomic-analysis). All markers were sequenced using the MiSeq technology from Illumina, which produced 250-base paired-end reads, except for the archaea marker, which was sequenced on the Illumina HiSeq platform, generating 100-base paired-end reads. In silico analysis showed that shorter archaea amplicons did not compromise ability to identify archaea at species level.

### Sequence analysis and taxonomic assignment

Alignment of paired-end reads, sample assignment and removal of sequences with ambiguous nucleotides and sequences of lengths outside the empirical sequence length distribution were performed with the OBITools software suite. Sequences were deposited in Dryad database under accession number http://dx.doi.org/10.5061/dryad.1b07d. Sequences were clustered into operational taxonomic units (OTU) at 97% similarity using UCLUST [31] and filtered for chimeric reads using ChimeraSlayer (bacteria, protozoa and archaea) or UCHIME (fungi) as implemented in QIIME pipeline v1.7.0 [32]. Taxonomy was assigned using the BLAST method [33]. Bacterial OTUs taxonomy was assigned using the Greengenes 12_10, archaeal—RIM-DB database [34], that of ciliate protozoa using the SILVA 18S database [35] and anaerobic fungi were assigned using a curated fungal ITS reference database [36] kindly provided by AgResearch Ltd (Palmerston North, New Zealand). Singleton OTUs were removed and the data from each sample were rarefied to the similar sequencing depth prior to further analyses using QIIME. Pairwise taxonomy comparisons were performed by computing Pearson correlation coefficients as implemented in QIIME. In total, 20 rumen-bolus-buccal swab-and faecal samples collected from the same animals at the same time were compared. Scatter plot analysis was done using R v2.15.0 [37].

For creating microbial co-occurrence networks in rumen and alternative sampling sites, the SparCC microbial association network inference tool [38] was used to calculate correlation coefficients between all bacteria, archaea, ciliate protozoa and fungi at the genus or the deepest identifiable taxonomic classification level. Correlations were derived where X = (x_ij), i,j = 1,2,…,m is the resulting correlation matrix with x_ij being the pair wise correlation between microbes i and j and m being the total amount of compared microorganisms. An adjacency matrix A = (a_ij), i,j = 1,2,…,m was determined where a_ij = 1, if x_ij> = 0.25, a_ij = -1, if x_ij < = -0.25 and a_ij = 0 else. Once determined, the adjacency matrix was used to construct a co-occurrence network, where each node represents a taxon while the edges between the nodes represent positive/negative correlations between taxa. Communities within the networks were identified by applying the leading eigenvector [39] method using the R-Package 'igraph' [40]. The pairwise similarities between the community structures of networks have been evaluated for each treatment and sampling site separately using the adjusted rand index [41] and visualized as a heatmap, where brighter colours refer to larger rand values indicating closer similarity.

## Results

In total, 80 samples, collected from four sampling sites, the rumen, buccal fluid, bolus and faeces were compared. Samples were collected from 5 lactating cows used in a 5 × 5 Latin Square with 28-d experimental periods to evaluate 5 experimental diets. Treatments comprised total mixed rations based on grass silage containing no additional fat (CO) or supplemented with methyl myristate (MA), rapeseed oil (RO), safflower oil (SO) or linseed oil (LO). Diets were formulated to induce changes in rumen microbial populations to provide a robust test of the suitability of sampling proxies.

### qPCR analysis of 16S and 18S rRNA genes from ruminal, buccal, bolus and faecal samples

**Fig 1:**
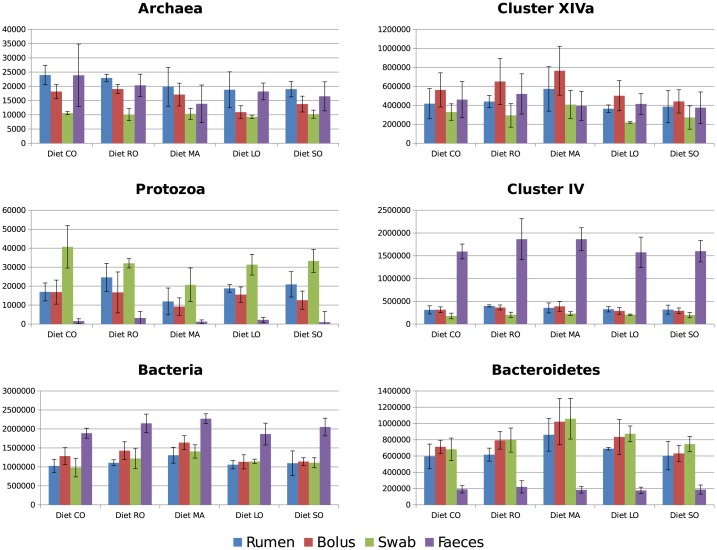
qPCR of 16S and 18S rRNA genes in buccal swabs, regurgitated digesta (bolus), ruminal digesta and faeces. Samples collected from lactating cows fed a grass silage based diet containing no additional fat (CO) or supplemented with 50 g/kg diet dry matter of methyl myristate (MA), rapeseed oil (RO), linseed oil (LO) or safflower oil (SO). Results are expressed as copy number per ng of extracted DNA. Error bars represent SD, n = 4 per treatment, except for four buccal samples, one each from CO, RO, LO and SO, from which satisfactory amplification was not obtained.

The abundance of different microbial groups was compared by qPCR across treatments and sample types (Fig 1; Tables B and C in S1 Text). Faecal samples differed markedly from corresponding ruminal samples, in that although archaea were present at a similar abundance, protozoa were virtually absent, and total bacteria were higher. *Clostridium* Cluster IV was on average 5× more abundant in faeces than in ruminal digesta. Bacteroidetes showed a correspondingly lower abundance.

Buccal swab samples contained similar abundances of total bacteria to ruminal samples, but the proportion of Bacteroidetes tended to be higher and *Clostridium* Clusters IV and XIVa lower. Archaea were 0.48× as abundant in swab samples compared to ruminal samples. Protozoal 18S rRNA abundance appeared to be 1.7× higher in swab samples.

Samples from the bolus of regurgitated digesta were generally closer in profile to ruminal digesta samples removed *via* the ruminal fistulae. Archaea were 0.80×, protozoa were 0.75× and total bacteria were 1.22× the abundance in ruminal digesta, although differences were not statistically significant due to high variability between samples. The proportions of the different classes of bacteria were similar.

No differences (*FDR* < 0.05) were detected due to treatment in any of the sample types, except that ciliate protozoa tended to be decreased in rumen and oral samples by dietary MA supplements, with a compensatory increase in total bacteria.

### Microbial composition of different sampling sites by rRNA gene amplicon analysis

**Fig 2:**
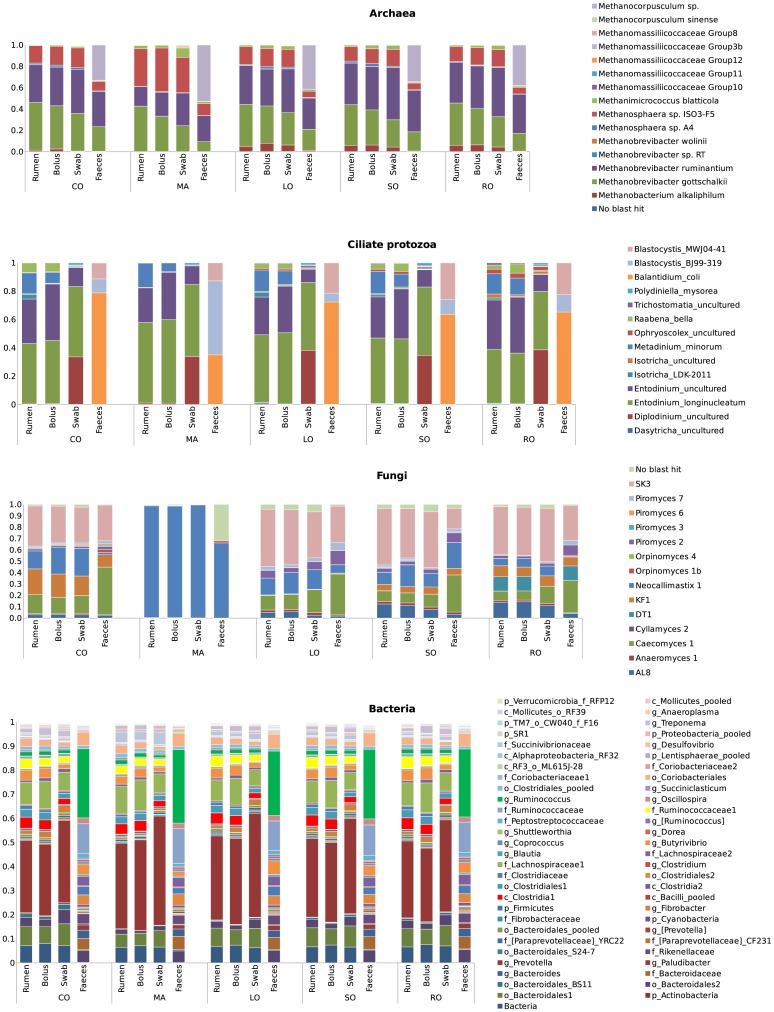
Relative abundance of bacteria, archaea, protozoa, and fungi based on amplicon sequencing of 16S-18S rRNA genes, and ITS1 sequences in rumen, bolus, buccal swabs and faeces. Samples collected from lactating cows fed total mixed rations based on grass silage containing no additional fat (CO), or supplemented with 50 g/kg dry matter of methyl myristate (MA), rapeseed oil (RO), safflower oil (SO) or linseed oil (LO). Data reported based on the mean of 4 animals per dietary treatment.

**Fig 3:**
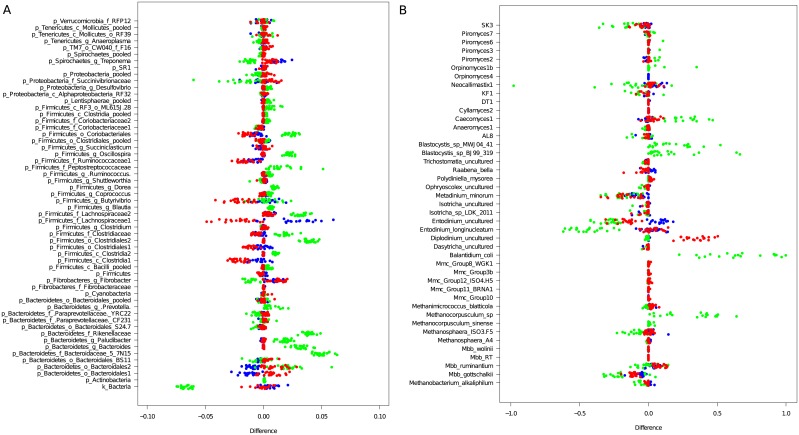
Changes in microbial abundance between rumen and the three alternative sampling sites. Each row represent genus-like microbial group for **a**) bacteria and **b**) archaea, ciliate protozoa, and fungi, while each dot represents individual cow. Differences were calculated as relative abundance in buccal swab minus abundance in rumen (red ○), faeces—rumen (green ○) and bolus—rumen (blue ○) based on n = 20 per sampling site.

Twenty rumen-bolus-buccal swab-faeces samples sets were collected for sampling site comparisons. In total, 7,305,504 high quality sequencing reads across all 4 major microbial groups (bacteria, archaea, ciliate protozoa and anaerobic fungi) were generated. The number of sequences assigned to each microbial group is reported in Table D in S1 Text. Microbiota composition is presented both as relative abundance in extracted DNA (Fig 2) and the difference in abundance relative to that determined in ruminal digesta (Fig 3; Fig A in S1 Text).

One hundred and four genus-like groups of bacteria were identified in the total dataset. Fifty of these had an average abundance above 0.5% in at least one of the treatments and sampling sites but only 16 could be identified to the genus level. The remaining 54 groups with average abundance below 0.5% were pooled at the lowest common taxonomic level. In total, they accounted for less than 5% of all sequencing reads. The archaeal community was represented by 9 genera consisting of 15 groups at the species level, while ciliate protozoa and fungal populations were represented by 14 groups at the genus level, respectively.

The bacterial communities in ruminal, bolus and buccal swab samples were remarkably similar to each other and affected little by treatment. The phylum Bacteroidetes was similar in rumen and bolus samples (ca. 45% of total sequences) but was 10% higher in buccal swabs and 20% lower in faeces. These differences were mainly related to the proportion of *Prevotella*, which was the most abundant bacterial genus in rumen-bolus-swab samples (Fig 2). The *Rikenellaceae* family and the phylum *Actinobacteria* were detected in faeces but not in other samples. Bolus samples also represented a close match to rumen samples in the abundance of Firmicutes (35%) while in faecal samples Firmicutes accounted for 70% of all sequences, with the *Ruminococcus* genus being the most abundant (Fig 2). Out of eight distinct bacterial groups within the *Lachnospiraceae* family, the most obvious differences among the sampling sites were related to a group of bacteria classified only at family level *(Lachnospiraceae1)*. This group was overrepresented in bolus, underrepresented in swabs and absent in faecal samples. Differences between rumen and swab samples were related to lower abundance of *Clostridia1*, *Ruminococcaceae1*, *Coriobacteriales* groups and *Butyrivibrio* genus in swabs, accounting for 1–2.5% lower abundances compared to ruminal digesta (Fig 3a).

In the archaeal communities, *Methanobrevibacter* dominated in rumen-bolus-swab samples but the proportion of *Mbb*. *gottschalkii* was underrepresented and *Mbb*. *ruminantium* overestimated in bolus and buccal swab samples compared to ruminal samples (Figs 2 and 3b). *Methanosphaera* was the second most common genus among archaea in these samples. In faeces, the *Methanocorpusculum* genus, not detected in rumen-bolus-swab samples, accounted for up to 53% of sequencing reads, with *Methanobrevibacter* and *Methanosphaera* genera being less abundant. Five distinct groups within the *Methanomassiliicoccaceae* family were detected in rumen-bolus-swab samples at similar abundance below 0.1% (Fig 3b). Diet had little influence on the archaeal community of the different sample types (Fig 2).

Ciliate protozoa composition in bolus samples matched closely rumen samples but differences were observed in ciliate taxon abundance. *Entodinium* was more abundant whereas *Isotricha* and *Metadinium minorum* were less abundant in bolus compared to rumen samples. Buccal swab samples contained a high proportion of *Diplodinium* (>33%) compared to rumen samples (<1%), whereas the proportion of uncultured *Entodinium* was lower by 16–23%. Furthermore, the relative abundance of *Metadinium minorum* and *Raabena bella* in buccal swabs was below 1% compared to 6–17% in rumen and bolus samples, while *Polydiniella mysorea* was detected in swabs (1–2%) but only in trace amounts in other sample types. No rumen specific protozoa were observed in faecal samples. The much lower abundance of faecal protozoa included the parasitic ciliate protozoa, *Balantidium coli* and *Blastocystis* sp. that were absent from rumen-bolus-swab samples. Treatment had little influence on the protozoal community of the different sample types (Fig 2).

Dietary lipid supplements had a profound effect on the fungal population composition, particularly methyl myristate (Fig 2). Apart from *Orpinomyces* 1b found only in faeces at an abundance below 3%, the composition of the fungal population in rumen-bolus-swab samples was similar. Differences were related to variation in abundance of *Caecomyces*1, KF1, *Neocallimastix*1 and SK3 fungal groups. The closest match was observed between rumen and bolus samples (Fig 3b).

### Correspondence analysis of microbial composition between sample types

**Fig 4:**
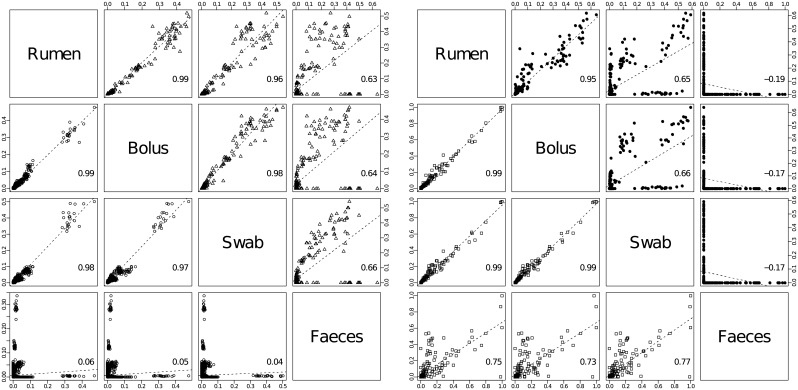
Scatter plot analysis of differences in relative abundance, estimated for each microbial taxon in all individual animals and across sample types, respectively. **a**) Each bacterial taxon in samples collected from all cows is represented as separate (○) in the lower triangle, while archaea are presented as (Δ) in the upper triangle; **b**) Anaerobic fungi are marked as (□) and ciliate protozoa as (●). The average Pearson correlation coefficient is indicated in the bottom-right corner of each diagram.

To evaluate how well the microbial communities in bolus, buccal swabs or faecal samples represented that in ruminal digesta, scatter plots were generated (Fig 4). In the scatter plots the relative abundances of every microbial taxon from each animal, representing all 3 non-invasive sampling sites, were compared with rumen samples. The strength of similarity between sampling sites was estimated as an overall Pearson correlation coefficient. The correlations derived indicated that bolus samples matched most closely the rumen composition for bacteria (r value = 0.99), while little overall correspondence was observed between faecal and rumen samples. Even though differences in abundance were observed among the more common bacterial groups in the buccal swab-rumen comparison, the overall correlation was high nonetheless (r value = 0.98). Patterns of similarity for archaea and anaerobic fungi among sampling sites was similar as for bacteria (Fig 4a and 4b). The largest difference between bolus and buccal samples compared with rumen digesta was for ciliate protozoa, while no relation to faecal protozoal composition was detected (Fig 4b).

### Microbial co-occurrence analysis

The potential of collecting bolus, faeces or buccal swabs as a reliable alternative to rumen sampling was examined through the generation of microbial association networks. Analysis was performed for each dietary treatment and on each sample type. Only correlations with SparCC |r| ≥0.25 were used for constructing the networks. Microbial communities identified within the networks of alternative sampling types were compared with rumen communities. Similarities of the networks are presented as a heatmap (Fig B in S1 Text). Microbial networks identified in bolus and swab samples did not offer an exact match to the network composition observed in rumen samples, while faecal microbial interactions were not comparable to the other sampling sites. Dietary treatments appeared to have an effect on the direct or indirect interactions between microbial taxa, with changes in response to MA showing the most distinct differences.

### Use of archaeal abundance as a biomarker of methane emissions

**Fig 5:**
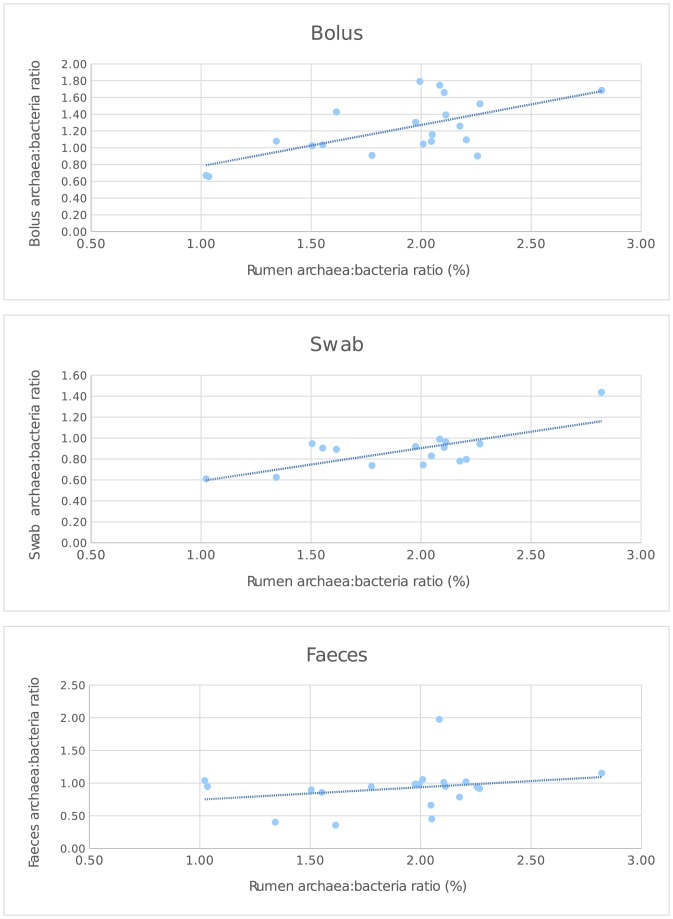
Archaeal abundance expressed as the archaea:bacteria ratio in different sample types in comparison with ruminal samples. Relationships are derived for individual animals from the dataset presented in Fig 1 based on n = 20 per sampling site, except for buccal swab samples, from which 16 were obtained. Regression equations relating the archaea:bacteria ratio in the rumen with alternative sample types are:- Bolus: Y = 0.491±0.1393 X + 0.290±0.2710, r2 = 0.376, n = 20, P = 0.0024 Swab: Y = 0.313±0.0828 X + 0.277±0.1621, r2 = 0.471, n = 16, P = 0.0020 Faeces: Y = 0.557±0.3377 X + 0.190±0.1736, r2 = 0.010, n = 20, P = 0.288.

The qPCR data for individual animals were used to calculate the ratio of abundance of archaea and bacteria, and the effects of dietary treatment and different sample types were compared. Inclusion of additional lipid in the dietary treatment decreased the relative proportions of archaea relative to bacteria in rumen samples from MA (*P* = 0.013) and LO (*P* = 0.033) treatments, with the responses to MA being the most pronounced, lowering the ratio of ruminal archaea:bacteria abundance by 36% compared to the control (Table E in S1 Text). The effects of lipid supplements were also evident in the other sample types, but the archaeal abundance was significantly lower in all other samples compared to ruminal digesta (Table E in S1 Text). When individual animals were compared across treatments, a closer correlation existed between ratio of archaea to bacteria in ruminal digesta with buccal swabs than bolus samples, while no association was evident in this ratio between rumen and faecal samples (Fig 5). However, neither buccal nor bolus samples showed a relationship that was close to unity with ruminal samples, indeed the slopes were much lower (0.491 and 0.313, respectively), although the positive intercepts did not differ (*P* > 0.10) from zero.

## Discussion

There is a pressing need to carry out large-scale ruminal microbiome analysis to generate a microbial phenotype for use as a trait in future animal selection for rumen function, including methanogenesis [6,42], feed conversion efficiency [6,42,43] and health [6,44,45]. Both methanogenesis and feed conversion efficiency have major implications on the environmental impact of ruminant livestock production [2,6]. The microbial phenotype may be defined as a simple ratio [46] or be far more complex based on microbial gene abundance [47]. The present paper reports the possibility to use oral samples as proxies for ruminal digesta, confirming the broad conclusions of a recent similar study in sheep [19]. While the communities were not identical, strong correlations were identified, indicating that the collection and analysis of oral samples could be used to predict the community structure in the rumen.

There have been many studies that examined the microbiota of the rumen and some that characterized the faecal community. Relatively few, however, have compared the two directly. Frey et al. [12], Michelland et al. [14] and Romero-Perez et al. [15] used restriction fragment analysis to obtain a fingerprint of each type of sample, showing marked differences in the gross structure of the two communities. More recently, deep sequencing methods were used [13], which demonstrated that there were much greater differences in detailed taxa than was revealed by restriction fragment analysis. The present analysis has parallels with the last study, while also comparing the eukaryotes and archaea.

The ruminal microbiota contains mainly bacteria, with lower numbers of archaea, anaerobic fungi and ciliate protozoa. The bacterial community was similar to those analysed previously, being dominated by Firmicutes and Bacteroidetes, the former mainly *Clostridium* Cluster XIVa and the latter mainly *Prevotella* spp. [3,5]. The archaeal, fungal and protozoal communities also had similar profiles to those observed in published studies [48,49]. The ruminant faecal microbiota has been examined previously [12–15], with results similar to those presented here in that *Clostridium* Cluster IV is much more predominant in faeces than the rumen and that Bacteroidetes are less abundant. The anaerobic fungal species in faeces were similar to the rumen, at slightly different abundances. Protozoa were present at low abundance and were not ruminal species. They represented parasitic ciliates that live in the gastrointestinal tract of various species [50]. In spite of the similar abundance of methanogenic archaea in faeces and the rumen as determined by qPCR, the faecal archaeal population was dominated by *Methanocorpusculum* rather than *Methanobrevibacter*.

Similarly, no comparisons have been made previously regarding oral and ruminal samples, indeed no ruminant oral microbiota analysis using modern cultivation-independent analysis appears to have been published. Two types of oral sample were investigated, namely buccal fluid (swabs) and grab samples of regurgitated digesta (boluses). Both would be extensively contaminated by saliva, thus preventing an estimation of absolute microbial abundances. However, it was envisaged that the microbial communities might be similar to each other, at least on a proportional basis. It emerged that the bacterial community of bolus samples was more similar to the ruminal digesta than buccal swab samples, presumably due to a distinct gingival microbiota that mixes with ruminal microorganisms derived from regurgitated food. The apparently high abundance of ciliates detected in swabs using qPCR was unexpected, and it remains to be established if the qPCR picked up non-protozoal sequences or, as suggested by the sequence analysis, there is genuinely a high proportion of *Diplodinium* in buccal swabs compared to rumen samples, suggesting that *Diplodinium* colonises the mouth.

The suitability of buccal swabs or regurgitated digesta as an alternative to rumen sampling will depend on the microbial communities being investigated and the line of scientific enquiry. Typically, the influence of environment, specifically diet, on the composition and function of the ruminal microbiome [15,51–53] and direct or indirect associations with animal performance traits [54–56] have been investigated. In the present experiment, the basal ration was supplemented with various sources of fatty acids differing in chain length and degree of saturation and used as means to test the robustness of alternative proxies. Inclusion of lipid supplements in the diet affected microbial populations in rumen and in alternative oral samples similarly. Compared with the control, lipid supplements had no effect (*P* > 0.05) on overall ruminal bacterial, archaeal or protozoal communities, as confirmed by qPCR. However, sequencing data indicated that MA completely changed the fungal community relative to all other treatments, with lesser effects on archaea and protozoa. Dietary supplements of myristic acid are known to lower methanogenesis in a dose dependent manner in lactating cows [57].

Within the core microbiome of gut ecosystems, microbial species co-occurrence is beginning to be understood [5,58]. Ruminal microorganisms do not exist in isolation and network analyses of taxa interactions across complex and diverse communities may help to ascertain the functional roles of uncultured microorganisms. Here, rumen-bolus-swab-faecal samples were compared in building microbial co-occurrence networks. The similarity of networks in alternative sampling types was measured by calculating the adjusted rand index between the different cluster structures. The cluster structures themselves were directly derived from the community detection of the coexistence networks. The results indicate that taxa coexistence networks created from interactions between bacteria, archaea, ciliate protozoa and fungi in bolus and swab samples were not a complete match to the rumen, while faecal networks had little in common with the other sampling types. Similar composition but variance in the abundance of individual microbial groups in samples collected from different sites influences the strength of associations used in the network analysis. In addition, specific environments like the mouth seem to harbour a set of specific microorganisms that may influence co-occurrence results when compared to rumen composition.

Until now, most ruminal microbiota analysis has been descriptive rather than predictive, although major studies are under way to change the situation regarding predictive approaches for methane emissions and feed efficiency [43,54–56]. Here, qPCR enabled the calculation of the archaea:bacteria ratio, which can provide a first approximation to predict methane emissions by individual animals [46]. The archaea:bacteria ratio in oral samples was different from corresponding rumen samples, and the slopes and intercepts of relationships between both sampling sites were not close to unity. Nonetheless, the moderate correlation between archaea:bacteria ratio in buccal and rumen samples suggest potential for screening purposes. The buccal samples were more representative than bolus, despite the closer correspondence between the observed microbial communities with bolus rather than buccal samples. No correlation existed between the archaea:bacteria ratio in the rumen and faeces. Such observations indicate that measurements of the faecal microbiome have little value as a biomarker of the rumen microbial community.

## Conclusions

The present experiments add significantly to previous observations [19,59] comparing different methods of obtaining ruminal digesta from live ruminants. Although the taxa present in buccal and bolus samples are similar to rumen samples, relative abundance varies. Nonetheless, because the differences seem to be consistent across animals and treatments, the estimation of microbial communities in these alternative samples may be useful for predictive purposes, such as would be required for screening large animal cohorts or wild ruminants. In contrast, the microbial composition in faeces has no resemblance to ruminal digesta or value as a biomarker of rumen function.

## Supporting Information

S1 Text Supporting information, containing five Tables and two Figures. Table A. Sequences of primers used for qPCR and amplicon sequencing. Table B. Within-diet significances of sample type in qPCR analyses shown in Fig 1. Table C. Between-diet significances of qPCR analyses shown in Fig 1. Table D. Number of filtered high quality sequences and the average number of sequences obtained per individual animal for each sampling site and for each microbial group, respectively. Table E. Archaea:bacteria ratio in different sample types across treatments. Figure A. Scatter plot of changes in microbial abundance between the rumen and the three alternative sampling sites calculated for each cow and each taxa, respectively. Figure B. Heatmap of microbial co-occurrence network analysis. Supporting references.
(DOCX)

## References

[1] Pfeffer E, Hristov AN. Interactions between cattle and the environment: a general introduction In: Pfeffer E, Hristov AN, editors. Nitrogen and phosphorus nutrition of cattle. CABI Publishing; 2007 pp. 1–12.

[2] Morgavi DP, Forano E, Martin C, Newbold CJ. Microbial ecosystem and methanogenesis in ruminants. Animal. 2010; 4: 1024–1036. 22444607 10.1017/S1751731110000546

[3] Hess M, Sczyrba A, Egan R, Kim TW, Chokhawala H, Schroth G, et al. Metagenomic discovery of biomass-degrading genes and genomes from cow rumen. Science. 2011; 331: 463–467. 10.1126/science.1200387 21273488

[4] Brulc JM, Antonopoulos DA, Berg Miller ME, Wilson MK, Yannarell AC, Dinsdale EA, et al. Gene-centric metagenomics of the fiber-adherent bovine rumen microbiome reveals forage specific glycoside hydrolases. Proc Natl Acad Sci USA. 2009; 106: 1948–1953. 10.1073/pnas.0806191105 19181843 PMC2633212

[5] Kittelmann S, Seedorf H, Walters WA, Clemente JC, Knight R, Gordon JI, et al Simultaneous amplicon sequencing to explore co-occurrence patterns of bacterial, archaeal and eukaryotic microorganisms in rumen microbial communities. PloS One. 2013; 8(2): e47879 10.1371/journal.pone.0047879 23408926 PMC3568148

[6] McAllister TA, Meale SJ, Valle E, Guan LL, Zhou M, Kelly WJ, et al Use of genomics and transcriptomics to identify strategies to lower ruminal methanogenesis. J Anim Sci. 2015; 93: 1431–1449. 10.2527/jas.2014-8329 26020166

[7] Shen JS, Chai Z, Song LJ, Liu JX, Wu YM. Insertion depth of oral stomach tubes may affect the fermentation parameters of ruminal fluid collected in dairy cows. J Dairy Sci. 2012; 95: 5978–5984. 10.3168/jds.2012-5499 22921624

[8] Duffield T, Plaizier JC, Fairfield A, Bagg R, Vessie G, Dick P, et al Comparison of techniques for measurement of rumen pH in lactating dairy cows. J Dairy Sci. 2004; 87: 59–66. 14765811 10.3168/jds.S0022-0302(04)73142-2

[9] Lodge-Ivey SL, Browne-Silva J, Horvath MB. Technical note: bacterial diversity and fermentation end products in rumen fluid samples collected via oral lavage or rumen cannula. J Anim Sci. 2009; 87: 2333–2337. 10.2527/jas.2008-1472 19329475

[10] McLeod MN, Minson DJ. Large particle breakdown by cattle eating ryegrass and alfalfa. J Anim Sci. 1988; 66: 992–999. 3378956 10.2527/jas1988.664992x

[11] Kennedy PM. Effect of rumination on reduction of particle size of rumen digesta by cattle. Austr J Agric Res. 1985; 36: 819–828.

[12] Frey JC, Pell AN, Berthiaume R, Lapierre H, Lee S, Ha JK, et al Comparative studies of microbial populations in the rumen, duodenum, ileum and faeces of lactating dairy cows. J Appl Microbiol. 2010; 108: 1982–1993. 10.1111/j.1365-2672.2009.04602.x 19863686

[13] de Oliveira MNV, Jewell KA, Freitas FS, Benjamin LA, Totola MR, Borges AC, et al Characterizing the microbiota across the gastrointestinal tract of a Brazilian Nelore steer. Vet Microbiol. 2013; 164: 307–314. 10.1016/j.vetmic.2013.02.013 23490556

[14] Michelland RJ, Monteils V, Zened A, Combes S, Cauquil L, Gidenne T, et al Spatial and temporal variations of the bacterial community in the bovine digestive tract. J Appl Microbiol. 2009; 107: 1642–1650. 10.1111/j.1365-2672.2009.04346.x 19457023

[15] Romero-Perez GA, Ominski KH, McAllister TA, Krause DO. Effect of environmental factors and influence of rumen and hindgut biogeography on bacterial communities in steers. Appl Environ Microbiol. 2011; 77: 258–268. 10.1128/AEM.01289-09 21075877 PMC3019729

[16] Gill FL, Dewhurst RJ, Dungait JAJ, Evershed RP, Ives L, Li Ch-S, et al Archaeol—a biomarker for foregut fermentation in modern and ancient herbivorous mammals? Org Geochem. 2010; 41: 467–472.

[17] McCartney CA, Bull ID, Yan T, Dewhurst RJ. Assessment of archaeol as a molecular proxy for methane production in cattle. J Dairy Sci. 2013; 96: 1211–1217. 10.3168/jds.2012-6042 23261373

[18] McCartney CA, Bull ID, Dewhurst RJ. Chemical markers for rumen methanogens and methanogenesis. Animal. 2013; 7 Suppl 2: 409–417. 23739482 10.1017/S1751731113000694

[19] Kittelmann S, Kirk MR, Jonker A, McCulloch A, Janssen PH. Buccal Swabbing as a noninvasive method to determine bacterial, archaeal, and eukaryotic microbial community structures in the rumen. Appl Environ Microbiol. 2015; 81: 7470–7483. 10.1128/AEM.02385-15 26276109 PMC4592876

[20] European Union. Council Directive 86/609/EEC on the approximation of laws, regulations and administrative provisions of the Member States regarding the protection of animals used for experimental and other scientific purposes. Off J L 358, 1–28 (1986). 20397315

[21] Yu Z. T. & Morrison M. Improved extraction of PCR-quality community DNA from digesta and fecal samples. Biotechniques 36, 808–812 (2004). 15152600 10.2144/04365ST04

[22] Maeda H, Fujimoto C, Haruki Y, Maeda T, Kokeguchi S, Petelin M, et al Quantitative real-time PCR using TaqMan and SYBR Green for *Actinobacillus actinomycetemcomitans*, *Porphyromonas gingivalis*, *Prevotella intermedia*, tetQ gene and total bacteria. FEMS Immunol Med Microbiol. 2003; 39: 81–86. 14557000 10.1016/S0928-8244(03)00224-4

[23] Fuller Z, Louis P, Mihajlovski A, Rungapamestry V, Ratcliffe B, Duncan AJ. Influence of cabbage processing methods and prebiotic manipulation of colonic microflora on glucosinolate breakdown in man. Br J Nutr. 2007; 98: 364–372. 17403273 10.1017/S0007114507709091

[24] Ramirez-Farias C, Slezak K, Fuller Z, Duncan A, Holtrop G, Louis P. Effect of inulin on the human gut microbiota: stimulation of Bifidobacterium adolescentis and Faecalibacterium prausnitzii. Br J Nutr. 2009; 101: 541–550. 10.1017/S0007114508019880 18590586

[25] Hook SE, Northwood KS, Wright ADG, McBride BW. Long-term monensin supplementation does not significantly affect the quantity or diversity of methanogens in the rumen of the lactating dairy cow. Appl Environ Microbiol. 2009; 75: 374–380. 10.1128/AEM.01672-08 19028912 PMC2620707

[26] Sylvester JT, Karnati SKR, Yu ZT, Morrison M, Firkins JL. Development of an assay to quantify rumen ciliate protozoal biomass in cows using real-time PCR. J Nutr. 2004; 134: 3378–3384. 15570040 10.1093/jn/134.12.3378

[27] Cole JR, Wang Q, Cardenas E, Fish J, Chai B, Farris RJ, et al The Ribosomal Database Project: improved alignments and new tools for rRNA analysis. Nucleic Acids Res. 2009; 37: D141–D145. 10.1093/nar/gkn879 19004872 PMC2686447

[28] Benjamini Y, Hochberg Y. Controlling the false discovery rate: a practical and powerful approach to multiple testing. J R Stat Soc Ser B. 1995; 57(1): 289–300.

[29] Riaz T, Shehzad W, Viari A, Pompanon F, Taberlet P, Coissac E. ecoPrimers: inference of new DNA barcode markers from whole genome sequence analysis. Nucleic Acids Res. 2011; 39: e145 10.1093/nar/gkr732 21930509 PMC3241669

[30] Boyer F, Mercier C, Bonin A, Le Bras Y, Taberlet P, Coissac E. OBITools Unix inspired software package for the DNA metabarcoding. Mol Ecol Resour. 2015; 15: 10.1111/1755-0998.12428 25959493

[31] Edgar RC. Search and clustering orders of magnitude faster than BLAST. Bioinformatics. 2010; 26: 2460–2461. 10.1093/bioinformatics/btq461 20709691

[32] Caporaso JG, Kuczynski J, Stombaugh J, Bittinger K, Bushman FD, Costello EK, et al QIIME allows analysis of high-throughput community sequencing data. Nat Methods. 2010; 7(5): 335–336. 10.1038/nmeth.f.303 20383131 PMC3156573

[33] Altschul SF, Gish W, Miller W, Myers EW, Lipman DJ. Basic local alignment search tool. J Mol Biol. 1990; 215: 403–410. 2231712 10.1016/S0022-2836(05)80360-2

[34] Seedorf H, Kittelmann S, Henderson G, Janssen PH. RIM-DB: a taxonomic framework for community structure analysis of methanogenic archaea from the rumen and other intestinal environments. PeerJ. 2014; 2:e494; 10.7717/peerj.494 25165621 PMC4137658

[35] Quast C, Pruesse E, Yilmaz P, Gerken J, Schweer T, Yarza P, et al The SILVA ribosomal RNA gene database project: improved data processing and web-based tools. Nucleic Acids Res. 2012; gks1219. 10.1093/nar/gks1219 PMC3531112 23193283

[36] Koetschan C, Kittelmann S, Lu J, Al-Halbouni D, Jarvis GN, Müller T, et al Internal transcribed spacer 1 secondary structure analysis reveals a common core throughout the anaerobic fungi (Neocallimastigomycota). PLoS ONE. 2014; 9: e91928 10.1371/journal.pone.0091928 24663345 PMC3963862

[37] R Development Core Team R: A language and environment for statistical computing. R Foundation for Statistical Computing, Vienna, Austria ISBN 3-900051-07-0. 2012 URL Available: http://www.R-project.org/.

[38] Friedman J, Alm EJ. Inferring Correlation Networks from Genomic Survey Data. PLoS Comput Biol. 2012; 8(9): e1002687 10.1371/journal.pcbi.1002687 23028285 PMC3447976

[39] Newman MEJ. Finding community structure using the eigenvectors of matrices. Phys Rev. 2006; E74: 036104. 10.1103/PhysRevE.74.036104 17025705

[40] Csardi G, Nepusz T. The igraph software package for complex network research, InterJournal Complex Systems. 2006; 1695–1703.

[41] Hubert L, Arabie P. Comparing Partitions. J Classif. 1985; 2: 193–218.

[42] Robinson DL, Goopy JP, Hegarty RS, Oddy VH, Thompson AN, Toovey VF, et al Genetic and environmental variation in methane emissions of sheep at pasture. J Anim Sci. 2014; 92: 4349–4363. 10.2527/jas.2014-8042 25149329

[43] Hegarty RS, Goopy JP, Herd RM, McCorkell B. Cattle selected for lower residual feed intake have reduced daily methane production. J Anim Sci. 2007; 85: 1479–1486. 17296777 10.2527/jas.2006-236

[44] Khafipour E, Li SC, Plaizier JC, Krause DO. Rumen microbiome composition determined using two nutritional models of subacute ruminal acidosis. Appl Environ Microbiol. 2009; 75: 7115–7124. 10.1128/AEM.00739-09 19783747 PMC2786511

[45] Rochfort S, Parker AJ, Dunshea FR. Plant bioactives for ruminant health and productivity. Phytochemistry. 2008; 69: 299–322. 17919666 10.1016/j.phytochem.2007.08.017

[46] Wallace RJ, Rooke JA, Duthie C-A, Hyslop JJ, Ross DW, McKain N, et al Archaeal abundance in post-mortem ruminal digesta may help predict methane emissions from beef cattle. Sci Rep. 2014; 4: 5892 10.1038/srep05892 25081098 PMC5376199

[47] Roehe R, Rooke JA, McKain N, Duthie C-A, Hyslop JJ, Ross DW et al Sire and breed effects on bovine methane emissions and feed efficiency correlate with the ruminal metagenome, indicating that microbial gene abundance can be used as a trait to breed more efficient livestock. BMC Genomics (in press)

[48] Gruninger RJ, Puniya AK, Callaghan TM, Edwards JE, Youssef N, Dagar SS, et al Anaerobic fungi (phylum Neocallimastigomycota): advances in understanding their taxonomy, life cycle, ecology, role and biotechnological potential. FEMS Microbiol Ecol. 2014; 90: 1–17. 10.1111/1574-6941.12383 25046344

[49] Williams AG, Coleman GS. The rumen protozoa In: Hobson PN, Stewart CS, editors. The Rumen Microbial Ecosystems. Springer Netherlands; 1997 pp. 73–139.

[50] Farthing MJ. Treatment options for the eradication of intestinal protozoa. Nat Clin Pract Gastroenterol Hepatol. 2006; 3: 436–445. 16883348 10.1038/ncpgasthep0557

[51] Tajima K, Aminov RI, Nagamine T, Matsui H, Nakamura M, Benno Y. Diet-dependent shifts in the bacterial population of the rumen revealed with real-time PCR. Appl Environ Microbiol. 2001; 67: 2766–2774. 11375193 10.1128/AEM.67.6.2766-2774.2001 PMC92937

[52] Franzolin R, St-Pierre B, Northwood K, Wright ADG. Analysis of rumen methanogen diversity in water buffaloes (Bubalus bubalis) under three different diets. Microb Ecol. 2012; 64: 131–139. 10.1007/s00248-012-0007-0 22286379

[53] Khejornsart P, Wanapat M. Diversity of rumen anaerobic fungi and methanogenic archaea in swamp buffalo influenced by various diets. J Anim Veterin Advances. 2010; 9: 3062–3069.

[54] Carberry CA, Kenny DA, Han S, McCabe MS, Waters SM. Effect of phenotypic residual feed intake and dietary forage content on the rumen microbial community of beef cattle. Appl Environ Microbiol. 2012; 78: 4949–4958. 10.1128/AEM.07759-11 22562991 PMC3416373

[55] Rius AG, Kittelmann S, Macdonald KA, Waghorn GC, Janssen PH, Sikkema E. Nitrogen metabolism and rumen microbial enumeration in lactating cows with divergent residual feed intake fed high-digestibility pasture. J Dairy Sci. 2012; 95: 5024–5034. 10.3168/jds.2012-5392 22916906

[56] Zhou M, Hernandez-Sanabria E, Guan LL. Assessment of the microbial ecology of ruminal methanogens in cattle with different feed efficiencies. Appl Environ Microbiol. 2009; 75: 6524–6533. 10.1128/AEM.02815-08 19717632 PMC2765141

[57] Odongo NE, Or-Rashid MM, Kebreab E, France J, McBride BW. Effect of supplementing myristic acid in dairy cow rations on ruminal methanogenesis and fatty acid profile in milk. J Dairy Sci. 2007; 90: 1851–1858. 17369226 10.3168/jds.2006-541

[58] Tap J, Mondot S, Levenez F, Pelletier E, Caron C, Furet J-P, et al Towards the healthy human intestinal microbiota phylogenetic core? Microb Ecol. 2009; 57: 580–581. 10.1111/j.1462-2920.2009.01982.x 19601958

[59] Henderson G, Cox F, Kittelmann S, Miri VH, Zethof M, Noel SJ, et al Effect of DNA extraction methods and sampling techniques on the apparent structure of cow and sheep rumen microbial communities. PLoS One. 2013; 8(9): e74787 10.1371/journal.pone.0074787 24040342 PMC3770609

