# Supplementary material for: Oral Samples as Non-Invasive Proxies for Assessing the Composition of the Rumen Microbial Community
Source: PLoS One. 2016 Mar 17;11(3):e0151220. doi: 10.1371/journal.pone.0151220 (PMC4795602; doi:10.1371/journal.pone.0151220)
Supplement: S1 Text — Table A. Sequences of primers used for qPCR and amplicon sequencing. Table B. Within-diet significances of sample type in qPCR analyses shown in Fig 1. Table C. Between-diet significances of qPCR analyses shown in Fig 1. Table D. Number of filtered high quality sequences and the average number of sequences obtained per individual animal for each sampling site and for each microbial group, respectively. Table E. Archaea:bacteria ratio in different sample types across treatments. Figure A. Scatter plot of changes in microbial abundance between the rumen and the three alternative sampling sites calculated for each cow and each taxa, respectively. Figure B. Heatmap of microbial co-occurrence network analysis. Supporting references. (DOCX) [file pone.0151220.s001.docx]

**Supporting Table A.** Sequences of primers used for qPCR and amplicon sequencing.

| Target group | Primer name/16S region | Nucleotide sequence | Main ruminal families/genera detected | Reference |
| --- | --- | --- | --- | --- |
| qPCR |  |  |  |  |
| All bacteria | UniF | GTGSTGCAYGGYYGTCGTCA | All bacteria | Maeda *et al*. [22] |
|  | UniR | ACGTCRTCCMCNCCTTCCTC |  | Maeda *et al*. [22] |
|  |  |  |  |  |
| All protozoa | 316f | GCTTTCGWTGGTAGTGTATT | All protozoa | Sylvester *et al.* [26] |
|  | 539r | CTTGCCCTCYAATCGTWCT |  | Sylvester *et al.* [26] |
|  |  |  |  |  |
| Archaea | Met630f | GGATTAGATACCCSGGTAGT | All archaea | Hook *et al.* [25] |
|  | Met803r | GTTGARTCCAATTAAACCGCA |  | Hook *et al.* [25] |
|  |  |  |  |  |
| Bacteroidetes | Bac303F | GAAGGTCCCCCACATTG | *Bacteroidaceae*: *Prevotella* and *Bacteroides* spp. | Bartosch *et al.* [60] |
|  | Bfr-Fmrev | CGCKACTTGGCTGGTTCAG |  | Ramirez-Farias *et al.* [24] |
|  |  |  |  |  |
| *Clostridium* | Clep866mF | TTAACACAATAAGTWATCCACCTGG | *Ruminococcaceae*: *Ruminococcus albus*, *R. flavefaciens* | Ramirez-Farias *et al.* [24] |
| Cluster IV | Clept1240mR | ACCTTCCTCCGTTTTGTCAAC |  | Ramirez-Farias *et al.* [24] |
|  |  |  |  |  |
| *Clostridium* | Erec482F | CGGTACCTGACTAAGAAGC | *Lachnospiraceae*: *Butyrivibrio*, *Pseudobutyrivibrio*, *Clostridium* | Rinttila *et al.* [61] |
| Cluster XIVa | Erec870R | AGTTTYATTCTTGCGAACG |  | Rinttila *et al.* [61] |
|  |  |  |  |  |
|  |  |  |  |  |
| Amplicon sequencing | Primer name | Nucleotide sequence | Region of amplification  16S rDNA V9  16S rDNA V9  16S rDNA V5-6  16S rDNA V5-6  18S rDNA  18S rDNA  ITS1  ITS1 | Reference |
|  |  |  |  |  |
| Archaea | ArchF | CCTGCTCCTTGCACACAC |  | This study |
|  | ArchR | CCTACGGCTACCTTGTTAC |  |  |
| Bacteria | BactF | GGATTAGATACCCTGGTAGT |  | This study |
|  | BactR | CACGACACGAGCTGACG |  |  |
| Protozoa | CiliF | CGATGGTAGTGTATTGGAC |  | This study |
|  | CiliR | GGAGCTGGAATTACCGC |  |  |
| Fungi | NeocF | TACCCTTTGTGAATTTGTT |  | This study |
|  | NeocR | ATCCATTGTCAAAAGTTGT |  |  |

**Supporting Table B.** Within-diet significances of sample type in qPCR analyses shown in Fig. 1. The comparisons are between rumen samples and the corresponding potential proxy sample. NS, *FDR* > 0.05.

| **Microbial group** | **Treatment** | **Rumen-Bolus** | **Rumen-Swab** | **Rumen-Faeces** |
| --- | --- | --- | --- | --- |
| Archaea | CO | NS | 0.026 | NS |
|  | RO | 0.039 | 0.019 | NS |
|  | MA | NS | NS | NS |
|  | LO | NS | NS | NS |
|  | SO | NS | 0.026 | NS |
| Protozoa | CO | NS | NS | 0.028 |
|  | RO | NS | NS | 0.028 |
|  | MA | NS | NS | NS |
|  | LO | NS | NS | 0.001 |
|  | SO | NS | NS | 0.033 |
| Bacteria | CO | NS | NS | 0.005 |
|  | RO | NS | NS | 0.019 |
|  | MA | NS | NS | 0.006 |
|  | LO | NS | NS | 0.032 |
|  | SO | NS | NS | 0.026 |
| Cluster XIVa | CO | NS | NS | NS |
|  | RO | NS | NS | NS |
|  | MA | NS | NS | NS |
|  | LO | NS | 0.026 | NS |
|  | SO | NS | NS | NS |
| Cluster IV | CO | NS | NS | 0.002 |
|  | RO | NS | NS | 0.032 |
|  | MA | NS | NS | 0.005 |
|  | LO | NS | NS | 0.026 |
|  | SO | NS | NS | 0.006 |
| Bacteroidetes | CO | NS | NS | 0.039 |
|  | RO | NS | NS | 0.005 |
|  | MA | NS | NS | 0.028 |
|  | LO | NS | NS | 0.001 |
|  | SO | NS | NS | 0.050 |

**Supporting Table C.** Between-diet significances of qPCR analyses shown in Fig. 1. NS, *FDR* > 0.05.

| **Microbial group** | **Treatment** | **Rumen** | **Bolus** | **Swab** | **Faeces** |
| --- | --- | --- | --- | --- | --- |
| Archaea | CO-RO | NS | NS | NS | NS |
|  | CO-MA | NS | NS | NS | NS |
|  | CO-LO | NS | NS | NS | NS |
|  | CO-SO | NS | NS | NS | NS |
|  | RO-MA | NS | NS | NS | NS |
|  | RO-LO | NS | NS | NS | NS |
|  | RO-SO | NS | NS | NS | NS |
|  | MA-LO | NS | NS | NS | NS |
|  | MA-SO | NS | NS | NS | NS |
|  | LO-SO | NS | NS | NS | NS |
| Protozoa | CO-RO | NS | NS | NS | NS |
|  | CO-MA | NS | NS | NS | NS |
|  | CO-LO | NS | NS | NS | NS |
|  | CO-SO | NS | NS | NS | NS |
|  | RO-MA | NS | NS | NS | NS |
|  | RO-LO | NS | NS | NS | NS |
|  | RO-SO | NS | NS | NS | NS |
|  | MA-LO | NS | NS | NS | NS |
|  | MA-SO | NS | NS | NS | NS |
|  | LO-SO | NS | NS | NS | NS |
| Bacteria | CO-RO | NS | NS | NS | NS |
|  | CO-MA | NS | NS | NS | NS |
|  | CO-LO | NS | NS | NS | NS |
|  | CO-SO | NS | NS | NS | NS |
|  | RO-MA | NS | NS | NS | NS |
|  | RO-LO | NS | NS | NS | NS |
|  | RO-SO | NS | NS | NS | NS |
|  | MA-LO | NS | NS | NS | NS |
|  | MA-SO | NS | NS | NS | NS |
|  | LO-SO | NS | NS | NS | NS |
| Cluster XIVa | CO-RO | NS | NS | NS | NS |
|  | CO-MA | NS | NS | NS | NS |
|  | CO-LO | NS | NS | NS | NS |
|  | CO-SO | NS | NS | NS | NS |
|  | RO-MA | NS | NS | NS | NS |
|  | RO-LO | NS | NS | NS | NS |
|  | RO-SO | NS | NS | NS | NS |
|  | MA-LO | NS | NS | NS | NS |
|  | MA-SO | NS | NS | NS | NS |
|  | LO-SO | NS | NS | NS | NS |
| Cluster IV | CO-RO | NS | NS | NS | NS |
|  | CO-MA | NS | NS | NS | NS |
|  | CO-LO | NS | NS | NS | NS |
|  | CO-SO | NS | NS | NS | NS |
|  | RO-MA | NS | NS | NS | NS |
|  | RO-LO | NS | NS | NS | NS |
|  | RO-SO | NS | NS | NS | NS |
|  | MA-LO | NS | NS | NS | NS |
|  | MA-SO | NS | NS | NS | NS |
|  | LO-SO | NS | NS | NS | NS |
| Bacteroidetes | CO-RO | NS | NS | NS | NS |
|  | CO-MA | NS | NS | NS | NS |
|  | CO-LO | NS | NS | NS | NS |
|  | CO-SO | NS | NS | NS | NS |
|  | RO-MA | NS | NS | NS | NS |
|  | RO-LO | NS | NS | NS | NS |
|  | RO-SO | NS | NS | NS | NS |
|  | MA-LO | NS | NS | NS | NS |
|  | MA-SO | NS | NS | NS | NS |
|  | LO-SO | NS | NS | NS | NS |

**Supporting Table D.** Number of filtered high quality sequences and the average number of sequences obtained per individual animal for each sampling site and for each microbial group, respectively.

| **Microbial group** | **Sample type** | **Amplicon length (bp)** | **High quality sequences** | **Average no of sequences per sample** | **Rarefaction depth** |
| --- | --- | --- | --- | --- | --- |
| Bacteria | Rumen | 256 | 325813 | 20363 | 7700 |
|  | Bolus |  | 383329 | 23484 | 7700 |
|  | Buccal swab |  | 327950 | 20313 | 7700 |
|  | Faeces |  | 194600 | 10132 | 7700 |
| Archaea | Rumen | 88 | 666769 | 40913 | 5900 |
|  | Bolus |  | 805842 | 51181 | 5900 |
|  | Buccal swab |  | 744580 | 43923 | 5900 |
|  | Faeces |  | 283303 | 8151 | 5900 |
| Ciliate protozoa | Rumen | 225 | 397830 | 24652 | 5700 |
|  | Bolus |  | 408823 | 25300 | 5700 |
|  | Buccal swab |  | 424341 | 26460 | 5700 |
|  | Faeces |  | 230661 | 16800 | 5700 |
| Anaerobic fungi | Rumen | 161 | 605581 | 38228 | 6000 |
|  | Bolus |  | 742138 | 47375 | 6000 |
|  | Buccal swab |  | 638223 | 40656 | 6000 |
|  | Faeces |  | 125721 | 4117 | 1600-6000^1^ |

^1^ In faecal samples that did not reach 6000 sequencing reads per sample for fungi, total available number of sequences was used.

**Supporting Table E.** Archaea:bacteria ratio in different sample types across treatments

| Treatment^1^ | **Archaea:bacteria ratio, sum of bacteria, %** | | | |  | **P value** |
| --- | --- | --- | --- | --- | --- | --- |
|  | **Rumen** | **Bolus** | **Swab** | **Faeces** |  |  |
| Diet CO | 1.77 | 1.08 | 0.92 | 1.03 | Rumen vs Bolus | 0.002 |
| Diet RO | 1.59 | 1.09 | 0.79 | 0.80 | Rumen vs Swab | 0.002 |
| Diet MA | 1.09 | 0.79 | 0.61 | 0.61 | Bolus vs Swab | 0.056 |
| Diet LO | 1.35 | 0.71 | 0.72 | 0.86 |  |  |
| Diet SO | 1.86 | 1.45 | 0.93 | 1.09 |  |  |

^1^Refers to total mixed rations based on grass silage containing no additional fat (CO), or supplemented with 50 g/kg dry matter of methyl myristate (MA), rapeseed oil (RO), safflower oil (SO) or linseed oil (LO). Lipid supplements replaced concentrate ingredients.

**Supporting Figure A**. **Scatter plot of changes in microbial abundance between the rumen and the three alternative sampling sites calculated for each cow and each taxa, respectively.** Each row represents the most abundant genus-like microbial group of bacteria, while each dot represents an individual cow. Differences in relative abundances were calculated as buccal swab - rumen (red dots), faeces - rumen (green dots) and bolus - rumen (blue dots).

**Supporting Figure B. Heatmap of microbial co-occurrence network analysis** between rumen samples and all three alternative sample types (B=bolus, S=buccal swab, F=faeces) collected simultaneously from individual cows (n = 20). Networks were constructed for each treatment (CO, LO, MA, RO and SO) separately. In the heatmap the brighter colours refer to larger rand values, indicating closer similarity of co-occurrence networks between sample types. Treatments comprised total mixed rations based on grass silage containing no additional fat (CO), or supplemented with 50 g/kg dry matter of methyl myristate (MA), rapeseed oil (RO), safflower oil (SO) or linseed oil (LO).


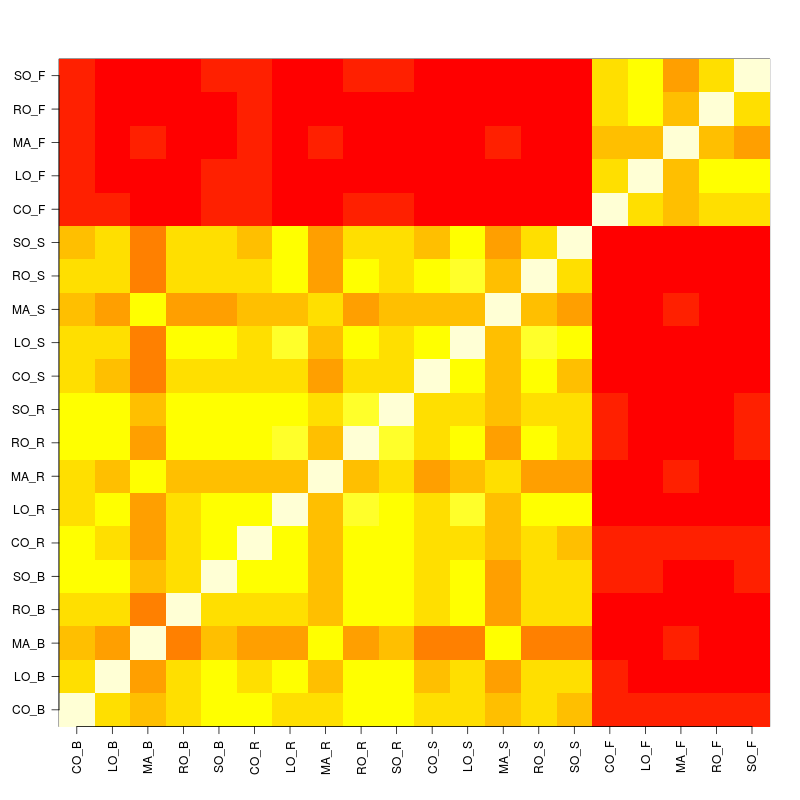


**Supporting references**

60. Bartosch S, Fite A, Macfarlane GT, McMurdo ME. Characterization of bacterial communities in faeces from healthy elderly volunteers and hospitalized elderly patients by using real-time PCR and effects of antibiotic treatment on the fecal microbiota. Appl Environ Microbiol*.* 2004; 70**:** 3575-3581.

61. Rinttila T, Kassinen A, Malinen E, Krogius L, Palva A. Development of an extensive set of 16S rDNA-targeted primers for quantification of pathogenic and indigenous bacteria in faecal samples by real-time PCR. J Appl Microbiol. 2004; 97: 1166-1177.
